# Common biological processes and mutual crosstalk mechanisms between cardiovascular disease and cancer

**DOI:** 10.3389/fonc.2024.1453090

**Published:** 2024-11-20

**Authors:** Hanwei Gao, Zhongyu Chen, Yutong Yao, Yuquan He, Xin Hu

**Affiliations:** ^1^ Department of Cardiology, China–Japan Union Hospital of Jilin University, Jilin University, Changchun, Jilin, China; ^2^ CJUH-JLU-China iGEM Team, Jilin University, Changchun, Jilin, China

**Keywords:** cancer, cardio-oncology, cardiovascular disease, cardiovascular toxicity, reverse cardio-oncology

## Abstract

Cancer and cardiovascular disease (CVD) are leading causes of mortality and thus represent major health challenges worldwide. Clinical data suggest that cancer patients have an increased likelihood of developing cardiovascular disease, while epidemiologic studies have shown that patients with cardiovascular disease are also more likely to develop cancer. These observations underscore the increasing importance of studies exploring the mechanisms underlying the interaction between the two diseases. We review their common physiological processes and potential pathophysiological links. We explore the effects of chronic inflammation, oxidative stress, and disorders of fatty acid metabolism in CVD and cancer, and also provide insights into how cancer and its treatments affect heart health, as well as present recent advances in reverse cardio-oncology using a new classification approach.

## 1 Introduction

Despite the substantial advancements in the prevention and treatment of cardiovascular disease (CVD) and cancer, these two conditions still claim millions of lives every year, and they remain the primary causes of early mortality worldwide (1). Traditionally, cancer and CVD have been considered distinct pathological conditions, the only connection being that cancer treatments can lead to cardiotoxicity and an increased risk of cardiac-related complications. However, clinical data suggest that patients with cancer have an increased probability of developing cardiovascular disease and that patients with cardiovascular disease are also more likely to develop cancer, indicating that there is a mutual relationship between the two diseases (2). Most researchers have focused primarily on how cancer and its treatment contribute to the development of CVD. However, the relationship between CVD and cancer may be more complex than previously thought, and the potential link between patients with CVD and their ensuing malignancies has not received widespread attention. A growing body of data suggests that patients with CVD have a higher risk of cancer compared to the general population. As a result, “reverse cardio-oncology” has begun to attract more attention. The exploration of the close connection between cancer and CVD is expected to increase the understanding of the mechanisms underlying both the development of the two diseases and their mutual influences, thereby providing new avenues for improving the treatment and prognosis of patients.

**Figure 1:**
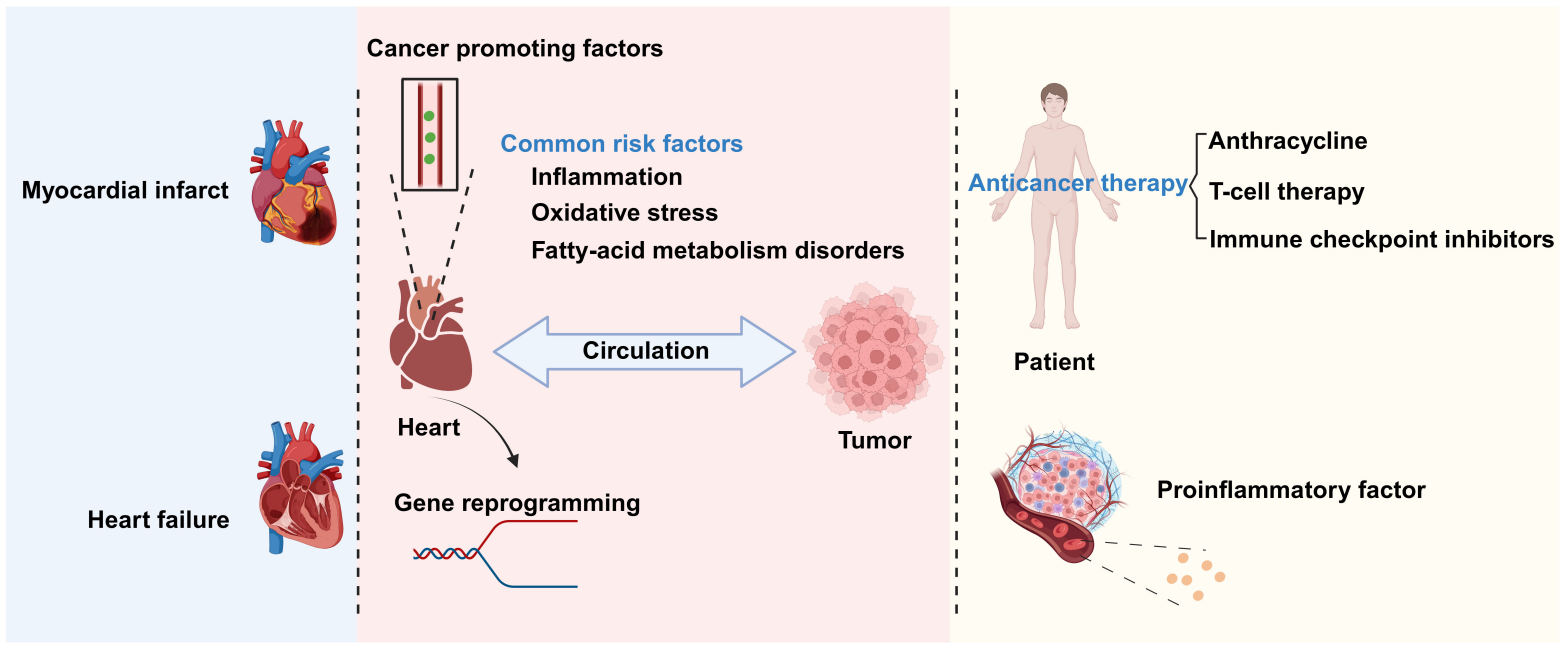
The relationship between cardiovascular disease (CVD) and cancer. There is a complex relationship between CVD and cancer. Biological processes shared by the two conditions include chronic inflammation, oxidative stress, and disrupted fatty acid metabolism. CVDs promote cancer occurrence and development through the secretion of factors and genetic reprogramming, among other mechanisms. Conversely, anticancer therapies and inflammatory factors secreted by cancer-associated cells can promote the progression of CVD. Created with BioRender.com.

There exists a complex relationship between CVD and cancer. These conditions share some biological processes and mutually influence each other’s pathogenesis and disease progression (3). Chronic inflammation, oxidative stress, and disordered fatty acid metabolism are common biological processes for CVD and cancer. The inflammatory cells and signaling molecules associated with chronic inflammation can increase the probability of patients developing CVD and tumors (4). Oxidative stress leads to increased intracellular oxidation reactions, which can damage cell structure and function, thereby promoting the occurrence and development of cancer and CVD. Meanwhile, fatty acid metabolism disorder can lead to elevated blood lipid levels, thus increasing the risk of atherosclerosis and cardiovascular events (5), as well as an excessive supply of fatty acids, which promotes tumor cell proliferation, invasion, and metastasis (6) (**Figure 1**).

The interaction between cancer and CVD is multifaceted. Tumors growing close to the heart, such as thymolipoma, may exert pressure on this organ, thereby affecting its normal function and leading to chest pain and difficulty breathing (7). In addition to direct mechanical pressure, anticancer treatments also have an impact on the heart. Chemotherapy and radiotherapy can damage cardiac tissue, leading to decreased heart function or the occurrence of CVD. In some instances, however, cancer treatment may exert ameliorative effects on CVD. At the same time, increasing evidence also indicates that CVD is associated with increased cancer incidence (8) through secreted factors and immune reprogramming, among other mechanisms (**Figure 1**).

In our review, we delve into the shared biological processes between cancer and CVD, and systematically outline the profound impact that cancer and its treatments may have on heart health. We specifically introduced the potential applications of anti-cancer therapy in the treatment of CVDs, as well as clinical cases of direct compression of the heart by tumors - these are areas that currently lack attention in research. In addition, we not only integrated the latest research findings on the increased risk of cancer in CVD, but also summarized existing studies and introduced new classification methods in order to provide a more comprehensive perspective for research in this field.

## 2 Shared biological processes between cancer and CVD

CVD and cancer share many risk factors, including diet, a sedentary lifestyle, aging, obesity, and smoking. Additionally, some signaling molecules and genetic pathways are crucial to the pathogenesis of both diseases (9), explaining their co-occurrence in certain situations. These observations highlight the importance of understanding the biological overlap between CVD and cancer. Chronic inflammation, oxidative stress, and disrupted fatty acid metabolism have been proposed as potential biological processes underlying the development of both CVD and cancer (9–11). For example, inflammation is a fundamental driver of atherosclerosis and can activate cells associated with atherosclerosis, such as endothelial cells and monocytes (12). Furthermore, inflammation and oxidative stress can promote atherosclerotic CVD and cancer by causing endothelial dysfunction (9). And endothelial dysfunction has been well documented in promoting cancer and CVD. Oxidative stress can directly damage cellular components by generating reactive oxygen species (ROS), which is another key factor in the development of CVD and cancer. Meanwhile, dysfunctional fatty acid metabolism leads to a dysregulation of saturated and unsaturated fatty acids, which is strongly associated with an increased risk of CVD, and cancer cells support their growth by altering their fatty acid metabolism, and fatty acid metabolites also promote cancer development by affecting immune responses and promoting inflammation.

### 2.1 Chronic inflammation

Inflammation is the body’s natural response to stimuli such as injury and infection. Chronic inflammation due to a variety of factors, including autoimmune diseases and chronic infections, can result in damage to the heart and other organs, thus increasing the risk of both CVD and cancer (13). Inflammation is a central driver of the development of atherosclerosis, which drives the progression of CVD by impairing vascular endothelial function, promoting plaque formation and its instability, and increasing the risk of thrombosis (14). Chronic inflammatory states play a key role in cancer development and evolution. Inflammatory cells in the tumor microenvironment and the cytokines they secrete not only promote cancer cell proliferation, survival, and metastasis, but also influence tumor escape from the host immune response and response to the effects of chemotherapy (13, 15).

#### 2.1.1 The promotive effect of chronic inflammation on cancer

**Figure 2:**
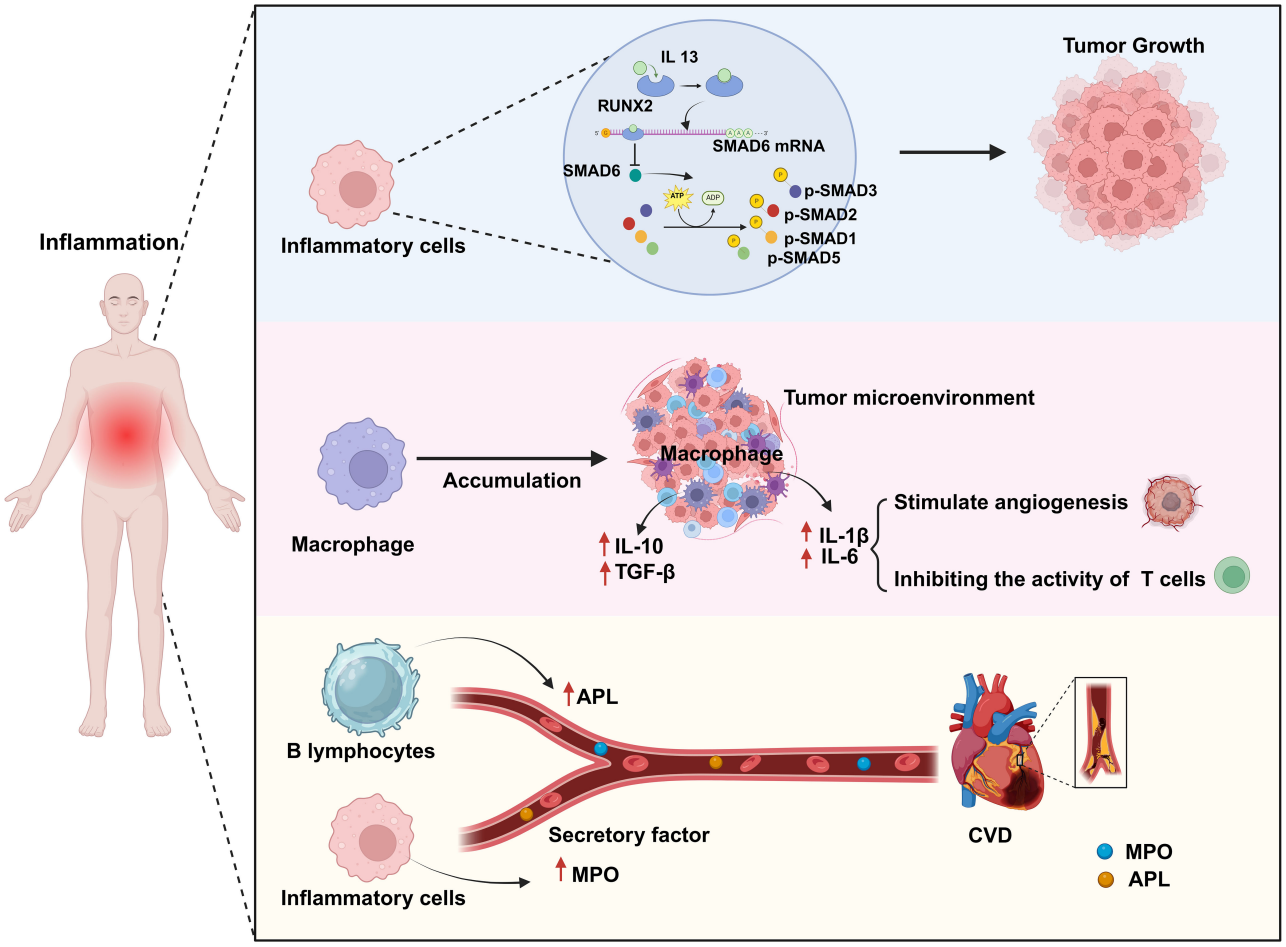
Chronic inflammation is a common biological process for both cardiovascular disease (CVD) and cancer. Chronic inflammation leads to an abnormal increase in the numbers of inflammatory cells and macrophages as well as in their activation. In this scenario, activated inflammatory cells promote tumor growth by activating a series of transcription factors (p-SMAD2/3, p-SMAD1/5). Simultaneously, activated macrophages promote angiogenesis by secreting factors such as IL-1β and IL-6 to provide nutrients to the tumor, while also secreting anti-inflammatory factors such as IL-10 and TGF-β to weaken T-cell attack on the tumor, thereby enhancing tumor growth and immune evasion. Additionally, some secreted cytokines (MPO, APL) can contribute to the formation of atherosclerosis, thus impairing endothelial cell function and increasing the risk of CVD. Created with BioRender.com.

Chronic inflammation can promote cancer occurrence and development through multiple mechanisms. The activation of different signaling pathways and cytokines during the inflammatory response enhances the pro-inflammatory reaction and facilitates the infiltration of macrophages, thereby promoting the proliferation and dissemination of cancer cells. Additionally, chronic inflammation can perturb the balance of the body’s immune system, increasing the likelihood of cancer cell survival. For example, Park et al. (16) demonstrated that chronic inflammation can lead to the dysregulation of anti-inflammatory factors, thereby promoting the progression of skin cancer. Skin and pancreatic epithelial cells exhibit significant levels of interleukin (IL)-33 expression under persistent inflammatory conditions. In the nucleus, IL-33 interacts with the transcription factor runt-related transcription factor 2 (RUNX2), thereby inhibiting the expression of the anti-inflammatory factor suppressor of mothers against decapentaplegic 6 (SMAD6). Consequently, there is an increase in the concentrations of p-SMAD2/3 and p-SMAD1/5, components of the transforming growth factor beta (TGF-β)/SMAD signaling pathway, which facilitates the growth, infiltration, and dissemination of skin cancer cells. Blocking TGF-β/SMAD signal transduction can inhibit IL-33-dependent epidermal cell proliferation and, consequently, the development of skin cancer (16) (**Figure 2**). In addition, cancer-induced inflammatory responses typically manifest as the aggregation of macrophages within the tumor microenvironment. When these macrophages infiltrate the tumor tissue, they release a range of cytokines that suppress the antitumor immune response of cytotoxic T cells while simultaneously encouraging the growth of new blood vessels to supply the tumor with oxygen and nutrients. Specifically, macrophages migrate to and accumulate in the tumor microenvironment in response to the release of chemical signals, such as cytokines and chemokines, by tumor cells. Once in the tumor tissue, macrophages become activated, which alters their function. Activated macrophages secrete cytokines, such as IL-1β and IL-6, which can stimulate angiogenesis and tumor cell proliferation. Furthermore, within the tumor microenvironment, most macrophages exhibit the M2 phenotype, which is characterized by the expression of several anti-inflammatory molecules, including IL-10, TGF-β, and arginase 1. This results in the establishment of an immunosuppressive microenvironment that promotes the growth and dissemination of tumors (17, 18) (**Figure 2**).

#### 2.1.2 The promotive effect of chronic inflammation on CVD

Chronic inflammation is strongly associated with the onset of CVD *via* a variety of mechanisms, including increasing the risk of atherosclerosis, the main underlying cause of CVD (19). For example, chronic inflammation can enhance myeloperoxidase (MPO) levels in the bloodstream, resulting in lipoprotein dysregulation; this can contribute to an increase in the occurrence of atherosclerosis and a reduction in the supply of nitric oxide, resulting in endothelial dysfunction, impaired vascular activity, and unstable atherosclerotic plaques, which further increase the risk of cardiovascular complications (20) (**Figure 2**). Autoimmune diseases can lead to chronic inflammation. Antiphospholipid syndrome (APS) is an autoimmune disorder defined by the presence of antiphospholipid antibodies (aPLAs), which can trigger inflammatory reactions through multiple mechanisms. They can affect the cardiovascular system by activating endothelial cells and monocytes, inducing the expression of cytokines and adhesion molecules, and promoting platelet aggregation and thrombin formation. Additionally, they can induce the transformation of macrophages into foam cells by promoting the generation of oxidized low-density lipoprotein (oxLDL)-β2GPI complexes, thus mediating thrombosis and the formation of atherosclerosis, and thereby increasing the risk of CVD (21). Conversely, inhibiting the inflammatory response can reduce the likelihood of developing this condition. Mammalian target of rapamycin (mTOR) signaling plays a significant role in the inflammatory mechanisms driving CVD. mTOR, a kinase, is involved in the regulation of basic cellular activities such as growth, proliferation, movement, energy consumption, and survival. Moreover, this kinase also controls inflammatory processes such as T-cell proliferation and activation, as well as monocyte and macrophage differentiation and development. Suppressing the mTOR signal can reduce the area of myocardial infarction (MI), improve cardiac function, and lower the risk of atherosclerosis. Medication targeting mTOR has demonstrated modest benefits in the management of CVD and cancer (22). In summary, the above observations indicate that chronic inflammation can increase the risk of CVD.

### 2.2 Oxidative stress

Oxidative stress is a critical factor in promoting the development of cancer and CVD. Free radicals and oxidants are generated during normal cellular metabolism and are essential for maintaining cellular functions and facilitating signal transduction. Nonetheless, when the production of these reactive species surpasses the cell’s antioxidant defense capacity, oxidative stress occurs. This results in cellular and tissue damage, which contribut*es* to the pathogenesis of various diseases. Oxidative stress-induced mitochondrial dysfunction and endothelial cell damage are key factors contributing to the development of CVD and cancer (23). In this process, the excessive accumulation of reactive oxygen species (ROS) causes extensive damage to key intracellular components, which include DNA, proteins and cell membranes. This damage not only affects normal cellular function, but also negatively affects cardiac cells in particular, leading to damage and apoptosis of these cells. Damage and apoptosis of cardiac cells are important factors in the decline of cardiac function and also provide favorable conditions for the development of cancer.

#### 2.2.1 The cancer-promoting effects of oxidative stress

**Figure 3:**
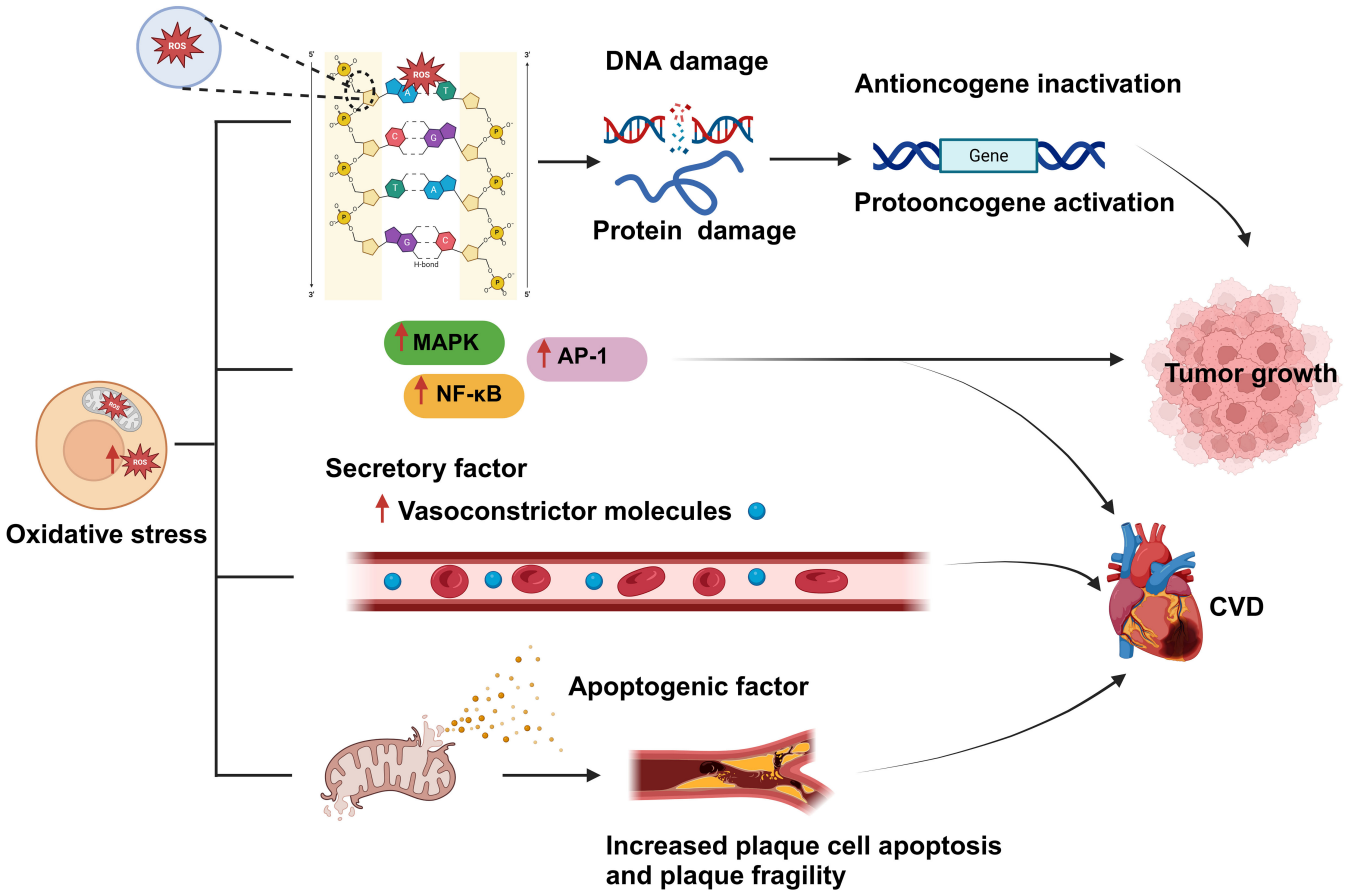
Oxidative stress is a shared biological process between cardiovascular disease (CVD) and cancer and is closely related to both conditions. An imbalance between reactive oxygen species (ROS) production and clearance by the body’s antioxidant defense system leads to the occurrence of oxidative stress. Excessive ROS production results in damage to cellular components, such as DNA and proteins; this, in turn, affects gene regulation, and leads to the inactivation of tumor suppressor genes and the activation of oncogenes, ultimately promoting tumor proliferation and development. In addition, oxidative stress can activate various protein kinases and transcription factors, such as MAPK, NF-κB, and activator protein 1 (AP-1), thereby promoting tumor cell proliferation, resistance to apoptosis, and the development of CVD. Moreover, excessive ROS generation can lead to the disruption of the mitochondrial membrane and the consequent release of proapoptotic factors, causing plaque cell rupture and apoptosis, which further exacerbates CVD development. Created with BioRender.com.

The excessive production of reactive oxygen species (ROS) significantly impacts signaling pathways and genomic integrity within the cellular milieu, thereby influencing the genesis and progression of cancer. Increased ROS levels disrupt cellular homeostasis and function, which is linked to the emergence of certain malignancies (24). ROS, including hydrogen peroxide, hydroxyl radicals, and superoxide anions, are highly reactive, and their overproduction can damage lipids, proteins, and DNA in cells (25). Specifically, ROS can interact with the sugar-phosphate backbone and nitrogenous bases of DNA, resulting in chromosomal abnormalities, strand breaks, and mutations. These genetic alterations can disrupt the regular control of the cell cycle, apoptosis, and DNA repair pathways. They may also lead to the activation of proto-oncogenes, which can enhance cell survival and proliferation, and the inactivation of tumor suppressor genes, which usually aid in controlling cell development and preventing cancer. The inhibition of tumor suppressor genes disrupts the regulation of normal cell growth, which can result in unchecked cell division and the creation of an environment that is favorable for tumor growth. Excessive ROS production can also result in protein damage and lipid oxidation, further exacerbating cell damage and death (24, 26, 27). Besides directly damaging the genome, ROS overproduction can also negatively affect multiple signaling pathways, thereby promoting tumor proliferation, invasion, and metastasis. For example, ROS can activate multiple protein kinases and transcription factors, such as mitogen-activated protein kinase (MAPK) and nuclear factor-kappa B (NF-κB), which can enhance cancer cell proliferation and resistance to apoptosis (28) (**Figure 3**).

#### 2.2.2 The promotive effect of oxidative stress on CVD

Oxidative stress can damage myocardial cells and disturb the functioning of the cardiovascular system and is thus one of the main causes of CVD development and incidence (29). For instance, aging, a primary risk factor for CVD (30), can be perceived as a process involving the accumulation of oxidative stress-induced damage. During aging, the ability of biological systems to neutralize oxidative stress gradually declines, leading to an imbalance between ROS generation and clearance, which increases ROS-mediated damage to cardiac tissue (31). Excessive cellular ROS production is mainly driven by mitochondrial metabolism and the NADPH oxidase (NOX) family of enzymes (32). ROS produced by mitochondria trigger the activation of certain transcription factors, including AP-1 and NF-κB, which, in turn, stimulate the inflammatory response (33) (**Figure 3**). ROS overproduction also weakens the antioxidant pathways, reduces the biological utilization of nitric oxide, and increases the generation of vasoconstrictive molecules, resulting in severe vascular dysfunction (34). Furthermore, the oxidative process can lead to mitochondrial membrane rupture, leading to the release of proapoptotic molecules, which increases the apoptosis of plaque cells and the fragility of plaques, stimulates smooth muscle cell proliferation, enhances leukocyte adhesion, and promotes inflammatory responses (35). All these mechanisms increase the risk of developing CVD.

### 2.3 Disruption of fatty acid metabolism

Fatty acid metabolism disorder refers to the abnormal synthesis, decomposition, and utilization of fatty acids in the body caused by genetics, diet, lifestyle, or other factors, which affects normal physiological functions and health status. Disorders of fatty acid metabolism play a crucial role in the development of CVD and cancer. In CVDs, abnormal fatty acid metabolism leads to insufficient energy supply, increased oxidative stress, intensified apoptosis, and inflammatory responses, which together promote the risk of atherosclerosis and heart disease (36). Meanwhile, excessive saturated fatty acid intake elevates bad cholesterol (LDL) in the blood, further increasing cardiovascular burden (37, 38).In contrast, short-chain fatty acids help reduce inflammation and protect cardiovascular health by regulating the gut microbiota (11). In the field of cancer, cancer cells promote tumor growth and metastasis by enhancing fatty acid synthesis and oxidation to meet their biosynthetic and energy needs (39). In addition, fatty acid metabolites can influence cancer development and progression by modulating immune responses and promoting inflammation (12). Therefore, the regulation and treatment of fatty acid metabolism is becoming an important research direction in the treatment of CVDs and cancer.

#### 2.3.1 Fatty acid metabolism disorders promote cancer growth and spread

**Figure 4:**
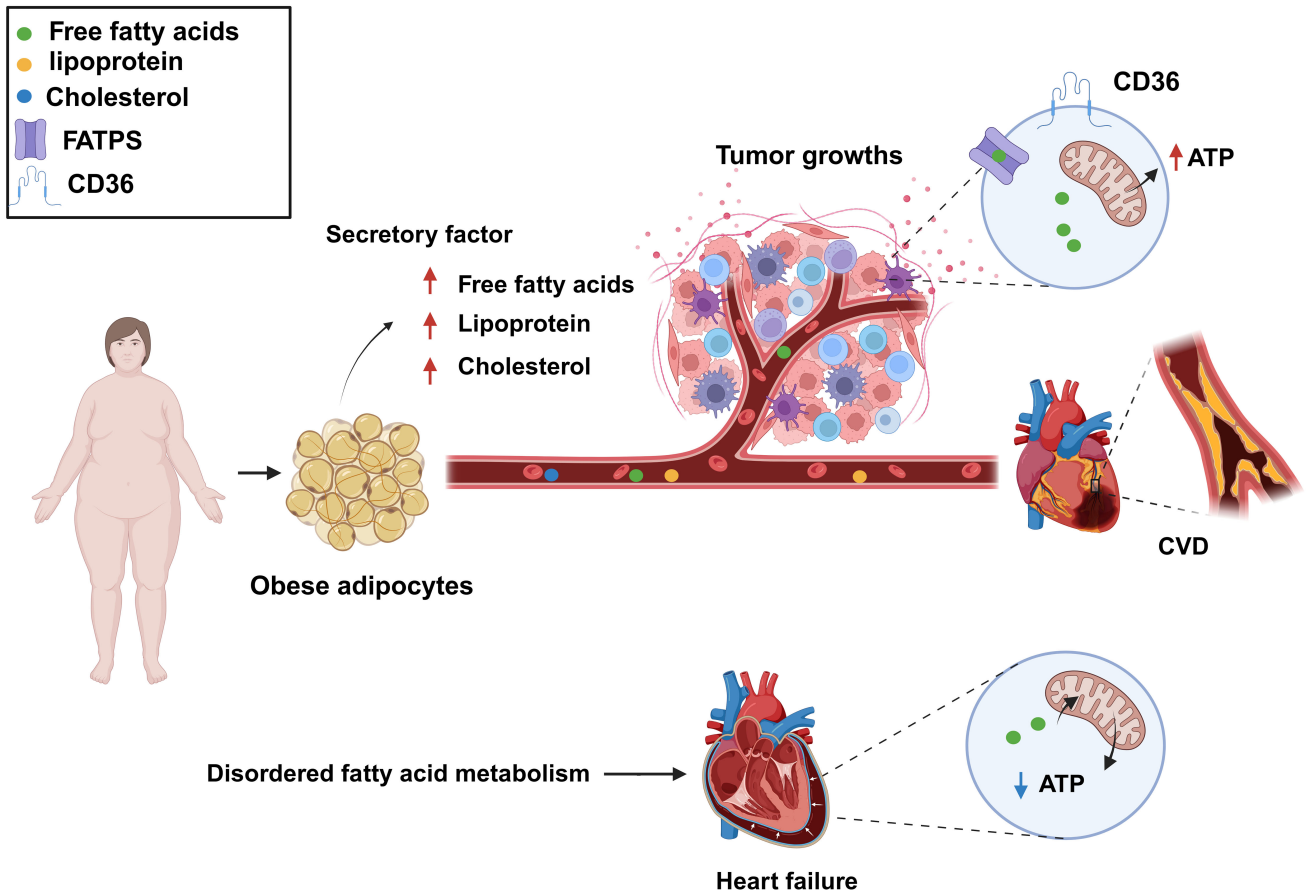
Dysregulated fatty acid metabolism is a common biological process for cardiovascular disease (CVD) and cancer and represents a key risk factor for the development of both conditions. Increased levels of free fatty acids, lipoproteins, and cholesterol in the blood can provide additional nutrients to cancer cells, meeting their growth demands, as well as lead to atherosclerosis. Fatty acid metabolism dysregulation can also result in decreased levels of fatty acids and adenosine triphosphate (ATP) production, resulting in impaired cardiac function. Created with BioRender.com.

Dysregulation of fatty acid metabolism affects the growth and dissemination of tumor cells by providing the energy and nutrients required for tumor survival and proliferation, as well as by fostering an environment that is conducive to tumor growth (40). Obesity is known to be associated with an increased risk of some cancers (41). During obesity, adipocytes release fatty acids, lipoproteins, hormones, and growth factors into the bloodstream, providing additional nutrients for cancer cells. Although cancer cells typically rely on aerobic glycolysis for energy, they can also use lipids and cholesterol as sources of energy. Cancer cells can obtain the necessary lipids by both absorbing exogenous lipids and synthesizing endogenous ones. They also utilize free fatty acids (FFAs) for mitochondrial oxidation and energy production, providing enough energy for their proliferation. FFAs are absorbed by cancer cells through specific proteins (FA translocase CD36, fatty acid transport proteins [FATPs]/SLC27A protein family, and fatty acid binding proteins [FABPs]). Cholesterol-rich lipoproteins are absorbed by cells to promote the formation of membrane microdomains, thereby mediating cancer occurrence and progression (11, 42) (**Figure 4**).

#### 2.3.2 Fatty acid metabolism disorders promote CVD

Fatty acid metabolism disorders, characterized by abnormal blood lipids and high cholesterol and triglyceride levels, promote atherosclerosis formation and thus are a significant influencing factor in the development of CVD. In addition, the dysregulation of fatty acid metabolism results in insufficient energy supply to myocardial cells, abnormal myocardial function, and myocardial ischemia (11). The heart is an energy-demanding organ and needs a substantial amount of adenosine triphosphate (ATP) to sustain its contractile function so that blood can be effectively pumped. Sufficient ATP production in cardiac muscle cells is crucial for maintaining heart function. Fatty acids and glucose are the primary sources of energy in the heart (43), with the former being the preferred energy fuel, generating approximately 70% of total ATP (44). Impaired fatty acid metabolism is a common cause of CVD. In cases of heart failure, fatty acid utilization is suppressed, leading to increased dependence on glucose as an energy source. This shift may reduce ATP production under the same energy demands, which affects the heart’s efficiency. Clinical studies have shown that ATP levels are reduced by over 30% in failing hearts, significantly impacting cardiac function. Fatty acid oxidation is also reduced in patients with heart failure, idiopathic dilated cardiomyopathy, and congenital heart disease (45–47). In certain situations, such as heart failure induced by aortic constriction, decreased fatty acid oxidation leads to a reduction in ATP generation (48) (**Figure 4**). Consequently, an imbalance in energy supply due to reduced cardiac fatty acid oxidation represents a major contributing factor to several heart conditions. Inflammation, excessive cell death, and oxidative stress can all result from abnormal fatty acid metabolism. Thus, targeting fatty acid metabolism is a primary focus of current CVD therapeutic strategies.

## 3 The effects of tumors on the heart

Tumors can affect the heart either directly or indirectly. For example, when a tumor grows near the heart, it may directly compress the heart as it increases in size, leading to impaired cardiac function (49). In addition to direct compression, some inflammatory factors secreted by cancer cells may promote CVD development. Meanwhile, anticancer treatments, such as chemotherapy and radiotherapy, may also exert toxic effects on the heart, leading to cardiac damage. However, in some cases, anticancer treatments may also have a positive impact on CVD treatment.

### 3.1 Direct effects of tumors on CVD

Tumors can have a direct impact on the heart. Tumor cells can directly press on the heart, leading to cardiac dysfunction, which can result in complications such as arrhythmia and heart failure. A clinical study conducted by Long et al. (50) found that hypodifferentiated breast cancer can compress the right ventricular outflow tract, which can cause cardiac damage. They reported a specific case of a 58-year-old woman diagnosed with poorly differentiated breast cancer whose diagnostic imaging at the time of admission to the hospital revealed that the tumor had spread to the brain, lungs, liver, and long bones of the lower extremities. Further chest CT scans showed that the tumor had invaded the sternum and left side of the mediastinum, causing external compression on the right ventricular outflow tract. Subsequent echocardiography confirmed increased pressure in the right ventricle, leading to the development of arrhythmias (50). Meanwhile, Weinberg et al. reported that compression of the aorta by metastatic squamous cell carcinoma can lead to aortic infarction. This finding was based on the case of a 69-year-old male patient who underwent left pneumonectomy for squamous cell carcinoma of the lung. Following surgery, the patient was readmitted to the hospital with unstable angina and continued to experience worsening angina symptoms over the next month before his untimely death. An autopsy revealed that the patient’s heart was almost completely encapsulated by metastatic cancer, with the tumor encircling the pulmonary trunk, the proximal ascending aorta, and the right and left atria. The left main pulmonary artery, in particular, was severely stenotic due to external compression by the tumor (51).

### 3.2 Indirect effects of tumors on CVD

As malignant tumors grow, they can indirectly affect cardiac function. For example, some cancers can trigger metabolic disorders through the secretion of biological factors that interfere with the normal functioning of the heart, which can lead to complications such as arrhythmia and CVD (52). In addition, anticancer therapy itself may adversely affect the heart, resulting in a range of complications such as cardiovascular lesions and myocarditis (53). However, in some cases, anti-cancer therapeutic measures can also be applied in the treatment of heart disease.

#### 3.2.1 Malignant tumors as risk factors for CVD

The continuous progress in cancer diagnosis and treatment methods has significantly improved the survival of numerous cancer patients. However, this progress has also revealed a new challenge, namely, that these patients are at a higher risk of death from CVD than from cancer recurrence (54). This is because cancer cells release a variety of inflammation-promoting factors and chemokines that not only damage endothelial cells but also increase microvascular permeability, leading to the leakage of pro-inflammatory factors (e.g., platelet-activating factor and tissue factor) into the extravascular space (55). This inflammatory process contributes to the aggregation of LDL and cholesterol particles in the vascular intima, leading to the formation of atherosclerotic plaques and accelerating the atherosclerotic process. Furthermore, cancer elevates the overall risk of coronary artery disease in cancer patients in addition to increasing thrombotic events (56, 57). Specifically, these newly formed plaques lead to the narrowing of the inner diameter of the blood vessels, which restricts blood flow and can cause symptoms of stable angina (58, 59). In addition, these plaques are prone to rupture, particularly those that have a thin fibrous cap covering their surface and whose centers are filled with thrombogenic material (thin-capped fibrous atherosclerotic plaques) (60, 61). The rupturing of a plaque or an increase in thrombus production may lead to blood vessel blockage, which can potentially trigger MI.

#### 3.2.2 Anticancer therapy causes cardiovascular toxicity

**Figure 5:**
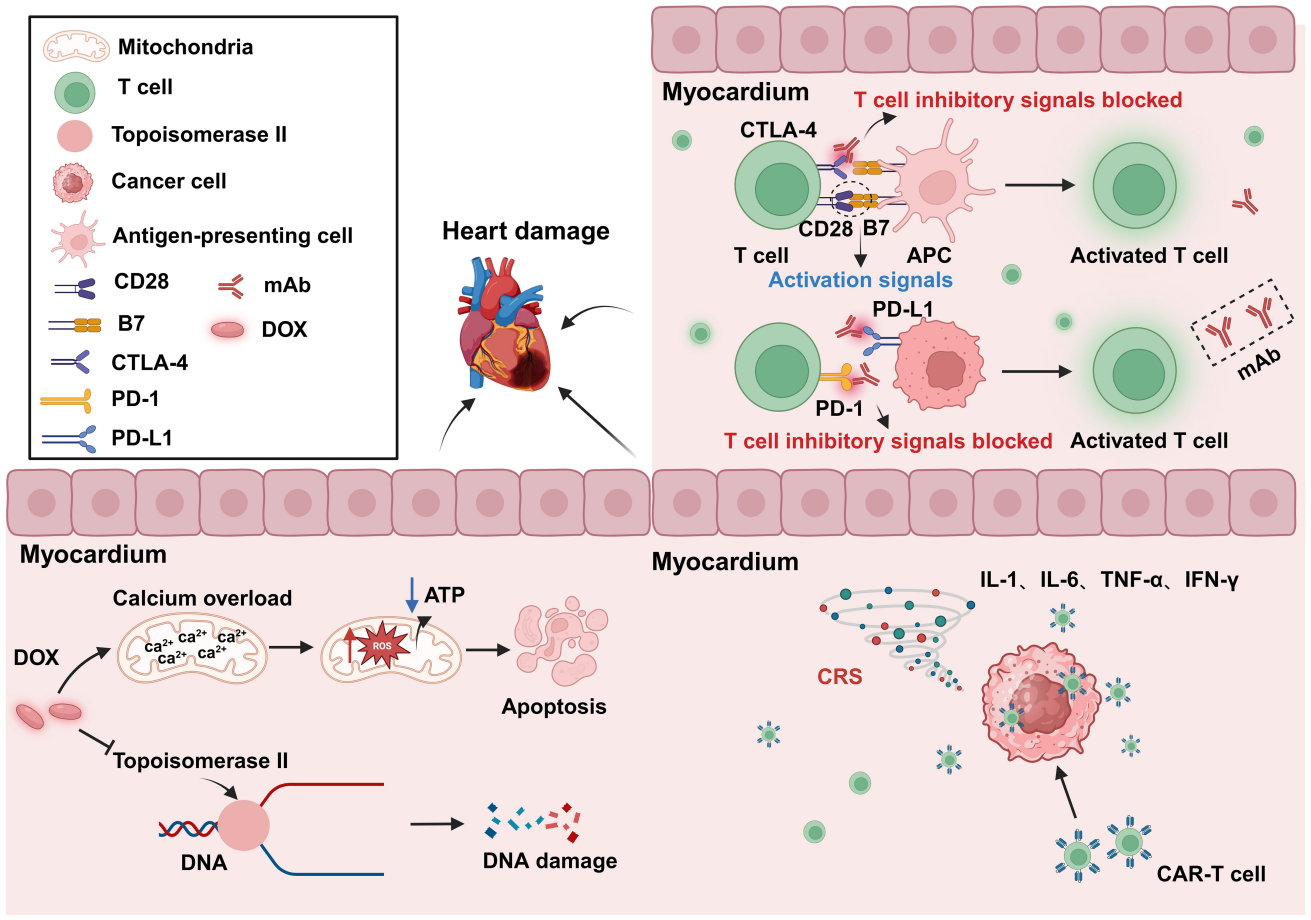
Cardiotoxicity induced by anticancer treatment. The use of the anticancer drug DOX can lead to DNA and mitochondrial damage, resulting in cytotoxicity. The inhibition of CTLA-4 and PD-1 can result in abnormal T-cell activation, leading to autoimmune myocarditis. CAR-T-cell therapy may cause CRS after tumor recognition, leading to the release of a large amount of proinflammatory factors, and resulting in cardiac toxicity. DOX, Doxorubicin; APC, Antigen-presenting cells; PD-1, Programmed cell death protein 1; PD-L1, Programmed cell death 1 ligand 1; CRS, Cytokine release syndrome; mAb, Monoclonal antibody. Created with BioRender.com.

In addition to its intended therapeutic function, anticancer treatments can also increase the risk of CVD in patients. Anticancer therapies include traditional chemotherapy and cancer immunotherapy, among others. The cardiotoxic effects of different types of chemotherapeutic agents and treatment intensities vary widely (62, 63), and are briefly summarized below (**Table 1**). For example, anthracycline-based chemotherapeutic agents, such as doxorubicin (DOX), are among the most widely used and best-characterized cancer treatments. However, DOX causes irreversible cardiotoxicity. It interacts with topoisomerase II, inhibiting its binding to DNA and causing DNA rupture (102, 103). It disrupts calcium homeostasis by directly affecting the calcium storage capacity of mitochondria through the activation of specific calcium channels that are sensitive to cyclosporine A (CsA). This results in calcium overload, leading to the impairment of mitochondrial function and apoptosis (104) (**Figure 5**). Cardiotoxicity is further exacerbated when DOX is used in combination with trastuzumab (105), which targets human epidermal growth factor receptor 2, and because cardiomyocytes express HER2, trastuzumab may affect cardiomyocyte function by interfering with the HER2 signaling pathway. Trastuzumab may also further damage cardiomyocytes by interfering with neuromodulin-1 signaling and mitogen-activated protein kinase (MAPK), phosphatidylinositol 3-kinase (PI3K)/Akt, and adhesion patch kinase (FAK)-dependent pathways (106). In addition, VEGF inhibitors play an important role in the treatment of a variety of cancers, and VEGF plays a key role in maintaining vascular integrity and function.VEGF inhibitors may affect the blood supply to the heart by decreasing angiogenesis, increasing vascular resistance and vascular stiffness (107). Immunotherapy for cancer involves activating or enhancing a patient’s immune system such that it can better detect and destroy cancer cells. Common immunotherapies include immune checkpoint inhibitors and chimeric antigen receptor (CAR)-T-cell therapy (108, 109). The use of cancer immunotherapy has revolutionized the treatment of various types of cancers associated with poor prognosis. However, it has also led to a wide spectrum of immune-related adverse events (110). First, the use of immune checkpoint inhibitors may lead to cardiotoxicity. Monoclonal antibodies (mAbs) targeting immune checkpoint molecules (cytotoxic T lymphocyte antigen-4 [CTLA-4], programmed death protein-1 [PD-1], and its ligand programmed death-ligand 1 [PD-L1]) have been used with marked success in the treatment of a variety of solid tumors (111–119). For various mAbs targeting PD-1 (nivolumab, pembrolizumab), PD-L1 (atezolizumab, durvalumab, avelumab), and CTLA-4 (ipilimumab), significant improvements in the treatment outcomes of melanoma and other cancers with poor prognosis have been observed (110, 120, 121). Nevertheless, it has been demonstrated that PD-1 and PD-L1 are also expressed in cardiomyocytes in both rodents and humans (99, 122–124) and that T-cell migration and proliferation are inhibited when PD-1 binds to its ligand, PD-L1. Meanwhile, CTLA-4 regulates the strength and duration of the immune response by competitively inhibiting the binding of CD28 to B7 molecules and inhibiting T-cell activation and proliferation. Animal studies have confirmed that the deletion of CTLA-4 and PD-1 can trigger autoimmune myocarditis (125–129). The use of CAR-T-cell therapy can also lead to cardiovascular toxicity, as evidenced by the possible triggering of cytokine release syndrome (CRS) after the recognition of tumor antigens by CAR-T cells. These release a variety of proinflammatory cytokines, including IL-1, IL-6, interferon-gamma, and tumor necrosis factor-alpha (TNF-α), which can trigger cytotoxic responses (130) (**Figure 5**). In addition, IL-6 not only has a direct impact on cardiac microvessels and cardiac contractility but can also induce the formation of an inflammatory environment and promote capillary leakage and microvascular dysfunction, further exacerbating the inflammatory response. This process may increase the generation of procoagulant factors (such as von Willebrand factor), leading to microvascular obstruction. Additionally, CRS may cause left ventricular dysfunction, ultimately leading to heart failure (130–142). Similarly, radiotherapy also contributes to cardiotoxicity; specifically, radiation may cause damage to cardiomyocytes, resulting in myocarditis or myocardial fibrosis, and prolonged or high doses of radiotherapy may affect the pumping function of the heart, leading to heart failure (143). In addition, radiotherapy may damage other substructures of the heart, such as the pericardium, coronary arteries, heart valves, and the conduction system, and these injuries may lead to pericarditis, coronary artery disease, valvular disease, and arrhythmias (144).

**Table 1:** The cardiotoxic effects of different types of chemotherapeutic drugs on the heart.

Drug	Therapeutic essence	Cardiotoxicity	Mechanism
Doxorubicin	Anthracycline (chemistry)	Left ventricular systolic dysfunction (LVSD) occurs in 5%–25% of patients and heart failure in 2%–6% of patients (64–70)	Anthracyclines induce the production of excess reactive oxygen-nitrogen species, leading to oxidative stress (71–78). Inhibits topoisomerase-2-beta (TOP2B) activity (79), induces DNA damage, and leads to mitochondrial defects
Trastuzumab	Target human epidermal growth factor receptor 2 (HER2)	The incidence of cardiotoxicity was reported to be 10% (a 0.10% decrease in the left ventricular ejection fraction), with 0.5% of these patients developing heart failure (80)	The precise cause of their cardiotoxicity has not been established. Nevertheless, it is understood that HER2 receptors within cardiomyocytes are pivotal in transmitting signals for growth and survival. Indeed, in a mouse model, the absence of HER2 receptors leads to dilated cardiomyopathy (81–84)
pertuzumab			
Sunitinib	Inhibits receptor tyrosine kinase activity; inhibits angiogenic pathways	The incidence of cardiotoxicity ranges between approximately 0.2% and 19% (64)	Sunitinib interacts with the tyrosine kinase domains of vascular endothelial growth factor (VEGF) receptor and platelet-derived growth factor receptor, affecting important physiological functions such as angiogenesis, cardiac contractility, diastole, and endothelial cell activity (85, 86)
Bevacizumab	Targets VEGF	According to estimates, 1.6% of cases include serious cardiac failure (87)	VEGF has an important role in angiogenesis. The inhibition of this pathway leads to impaired cardiac angiogenesis
Ponatinib	Multi-targeted kinase inhibitor targeting the breakpoint cluster region–Abelson 1 (BCR-ABL) gene	Can result in arterial blood clotting, venous thrombosis, and high blood pressure; on rare occasions, it may also result in LVSD (88, 89)	Has an inhibitory effect on VEGF and platelet-derived growth factor receptors
Bortezomib	Proteasome inhibitors	Patients can experience adverse effects such as heart failure, hypertension, and arrhythmia. In clinical trials in patients with multiple myeloma, the incidence of heart failure ranged from 3.8% to 40% (90–93)	Can inhibit proteasome activity in cardiomyocytes
kafizome			
Darafenib	v-Raf murine sarcoma viral oncogene homolog B1 (BRAF) and MEK inhibitors	Lead to hypertension and prolonged QT interval. According to a review of clinical trials, varying degrees of LVSD were observed in approximately 5.7% to 11.7% of patients (94)	The mechanisms responsible for cardiotoxicity induced by BRAF and MEK inhibitors remain unclear. However, studies conducted using animal models have underscored the critical importance of mitogen-activated protein kinase pathways in enhancing the growth, viability, and regeneration of cardiomyocytes (95–98)
verofinil			
encorefenib			
cobimetinib			
trametinib			
binimetinib			
Ipilimumab	Immune checkpoint inhibitor	The incidence of myocarditis ranges from approximately 0.27% to 1.14%. However, this myocarditis carries a risk of death of up to 50% (99–101)	May cause a range of immune-related toxic reactions

#### 3.2.3 Anti-cancer drugs have the potential to treat CVD

Anti-cancer drugs are known to exert adverse effects on CVD; however, recent studies have reported that, under certain circumstances, some anticancer drugs have shown therapeutic potential in CVD (145). Cilengitide is an anticancer drug that was originally developed as an antiangiogenic agent with a maximum tolerated dose of 5-50 mg/kg (146, 147). However, studies have found that at low doses, cilengitide has potential as a treatment for heart failure. One of the main causes of heart failure progression is coronary microvascular dysfunction and cardiomyocyte morphological and energetic maladaptation, implying that the course of heart failure can be slowed by increasing cardiac angiogenesis and boosting cardiomyocyte function. In healthy hearts, αvβ3 integrin expression is minimal, but in damaged hearts, it is highly expressed in endothelial cells and cardiomyocytes (148, 149). Heart failure can be effectively treated by targeting αvβ3 integrin. At low doses, cilengitide, although not an antagonist of αvβ3 integral protein, can enhance the activation and recycling of VEGFR2 by altering αvβ3 signaling (150). One study found that the administration of low-dose cilengitide decreased coronary angiogenesis and cardiomyocyte hypertrophy in a mouse model of post-surgical abdominal aortic constriction (145). In addition, CAR-T-cell therapy was found to have potential therapeutic value in a mouse model of induced cardiac fibrosis. It was shown that CAR-T-cell therapy selectively eliminates cardiac myofibroblasts that express fibroblast-activating protein (FAP), identified as a marker for this cell type. Cardiac fibrosis is the process by which extracellular matrix proteins are deposited between cardiomyocytes, leading to the remodeling and hardening of cardiac tissue. Fibrosis is a crucial factor in the etiology of various CVDs, including heart failure and MI. Fibroblasts are the main cell type involved in the fibrotic process. Employing a mouse model of cardiac fibrosis, the authors of this study showed that CAR-T-cell therapy targeting FAP attenuated cardiac fibrosis, thereby helping to maintain normal cardiac systolic and diastolic function. Even though these results were significant, more research is required to evaluate the potential clinical benefits of these novel therapeutic strategies (151).

## 4 The impact of CVD on cancer

**Figure 6:**
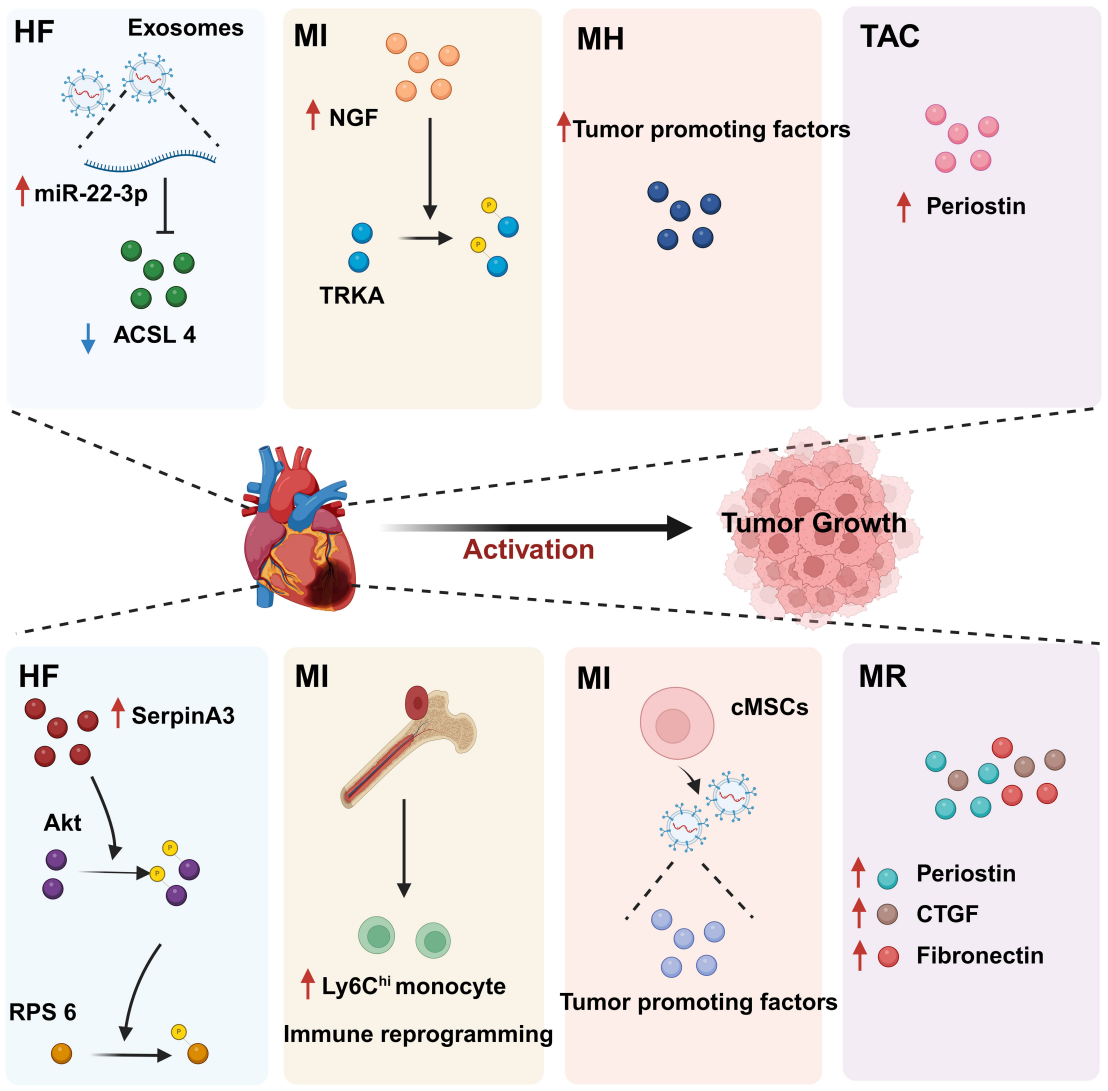
The regulatory effect of CVD on tumors. CVD can promote tumor progression through secreted factors and immune reprogramming. In individuals with heart disease, cardiac cells secrete large amounts of tumor growth-promoting factors, including proteins and miRNAs. In addition, CVD can contribute to tumor immune evasion through immune reprogramming. These phenomena suggest that there is a complex relationship between CVD and cancer. HF, Heart failure; MI, Myocardial infarction; MH, Myocardial hypertrophy; CVD, Cardiovascular disease; MR, Myocardial remodeling; TAC, Transverse aortic constriction. Created with BioRender.com.

We previously mentioned the impact of cancer on CVD, focusing on the effects of anticancer drugs on the heart, known as “oncocardiology”. However, how CVD influences cancer has been relatively understudied. Indeed, CVD may be an independent risk factor for cancer development. Both diseases are often present simultaneously in patients, leading to increased suffering and treatment limitations. Therefore, it is essential to uncover the molecular mechanisms underlying the link between CVD and cancer, which is crucial for understanding the key processes involved in the development of these diseases. We have found that CVD can regulate cancer progression through secreted factors and genetic reprogramming (152, 153) (**Figure 6**).

### 4.1 CVD promotes tumor proliferation and migration through secreted factors

**Figure 7:**
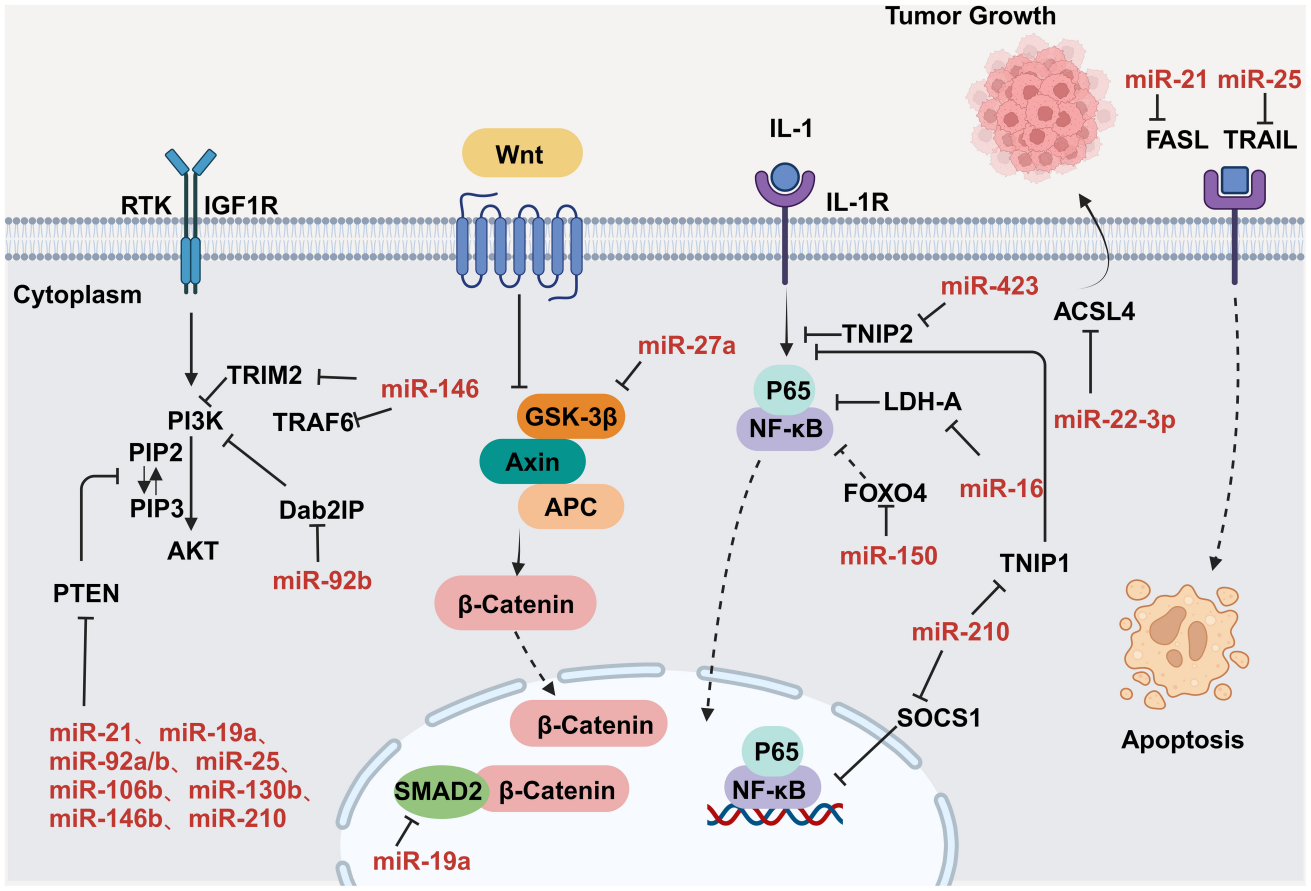
MiRNAs regulate tumor progression by modulating a variety of signaling pathways. Key nodal proteins involved in the respective pathways are shown in black; red text indicates exosomal miRNAs of circulating origin, which can be upregulated in tumor cells; solid black arrows indicate promotion or activation; line segments indicate inhibition; dashed arrowheads indicate multistep transfer or activation. Created with BioRender.com. Created with BioRender.com.

CVD can promote tumor proliferation and migration through secreted factors. Studies have shown that serpinA3 protein is upregulated in patients with heart failure as well as in APC^min^ mice, a line that is prone to colorectal cancer, in which infarction was induced. Additionally, serpinA3 upregulation resulted in increased phosphorylation of both AKT and its downstream target ribosomal protein S6 (rpS6), which contributed to colorectal cancer development (154) (**Figure 6**). In addition, several studies have found that post-MI cells secrete exosomes encapsulating tumorigenic factors that can target colorectal and lung cancer cells, thereby promoting tumor development. Specifically, cardiac mesenchymal stromal cells (cMSCs), essential for cardiac repair, remodeling, and fibrosis, were extracted from the hearts of MI and pseudo-MI model mice. After MI, the cMSCs secreted small exosomes containing a large number of tumorigenic factors such as periostin, osteoblastin, VEGF, IL-6, and TNF-α, in addition to tumorigenic microRNAs. These exosomes were observed to target lung and colorectal cancer cells and significantly promote tumor proliferation. Following exosome depletion, the MI-induced tumorigenic effect was significantly attenuated (155). In the previous, we focused on the association between MI and poor cancer prognosis. Next, we discuss whether early cardiac remodeling promotes cancer progression in the absence of MI. Avraham et al. (8) conducted a study in which they showed that early cardiac remodeling can enhance tumor progression in mice through the release of secreted factors. The authors first subjected C57B1/6 female mice to transverse aortic constriction (TAC) to induce cardiac hypertrophy, and subsequently implanted cancer cells into the animals. They observed increased cardiac dimensions and elevated expression of hypertrophic markers such as ANP, BNP, βMHC, and ACTA1 in the hearts of TAC-operated mice. Interestingly, there was no evidence of cardiac fibrosis in these animals. Despite displaying mild cardiac remodeling and hypertrophic characteristics without symptoms of heart failure, the TAC-operated mice developed larger tumors with higher proliferation rates and had a greater number of metastatic foci compared with untreated or sham-operated mice. Moreover, serum from TAC-operated mice was found to facilitate cancer cell proliferation *in vitro*. RNA-seq analysis revealed elevated levels of the extracellular matrix proteins periostin and CTGF in the heart and serum of mice after TAC; both of these proteins are known to influence cancer cell behavior, including proliferation, migration, and epithelial-to-mesenchymal transition. The upregulation of periostin and CTGF in the heart following TAC surgery further highlights the association between cardiac remodeling and enhanced tumor growth. This study underscores the role of early cardiac changes in promoting tumor progression (8). Meanwhile, Awwad et al. (156) established a mouse model of hypertension-dependent cardiac remodeling by infusing low doses of phenylephrine (PE) to induce chronic hypertension, followed by the injection of PyMT breast cancer cells *in situ*. Their study also demonstrated that early cardiac remodeling can promote cancer development. Echocardiography revealed no appreciable loss of heart function, despite the identification of genetic markers associated with cardiac remodeling, hypertrophy, and fibrosis. However, in mice administered PE, the tumors derived from PyMT cells were larger and had a higher proliferation rate than those observed in control animals. Additionally, compared with the controls, mice treated with PE showed higher serum levels of periostin, fibronectin, and CTGF. In line with this observation, serum from PE-treated mice enhanced the proliferation of cancer cells *in vitro* to a greater extent than serum derived from non-infused mice (156). Overall, these findings underscore that early cardiac remodeling before the occurrence of heart failure is sufficient to promote tumor growth (**Figure 6**). While these are examples of cardiac stress induced by major surgical interventions, it has been found that secreted factors can also promote tumor development in a mouse model of spontaneous cardiac hypertrophy. Awwad et al. (157) induced cardiac hypertrophy and cardiac dysfunction by expressing activating transcription factor 3 (ATF3) in mouse cardiomyocytes, which were then transplanted *in situ* with breast and lung cancer cells. The tumors that formed in ATF3 transgenic mice were larger and exhibited more prominent metastatic characteristics compared with those that formed in wild-type animals (157). Additionally, the levels of periostin, connective tissue growth factor (CTGF), and plasma cuprocyanin, all of which have significant roles in cancer progression, were elevated in these mice, as was that of serpinA3 protein, which promotes colorectal cancer development (153). Thus, it seems that *ATF3* transgene expression in cardiomyocytes initiates a harmful sequence of events that result in cardiac hypertrophy, fibrosis, and dysfunction. This cascade eventually leads to the secretion of tumorigenic factors by the heart, tumors, and other organs, which further exacerbates tumor growth and spread. This suggests that the expression of ATF3 in cardiac cells may contribute to the development and progression of cancer by promoting a cycle of detrimental interactions between the heart and tumors (153). In addition, CVD leads to altered blood miRNA expression profiles, which can adversely affect concomitantly occurring tumors (158, 159). This is due to the fact that tumors frequently exchange materials with their surroundings as they are highly heterogeneous tissues that are sensitive to blood circulation. Changes in the levels of CVD-linked miRNAs may impact the control of the tumor microenvironment, immune infiltration into the tumor, and metabolic reprogramming (160–162). Furthermore, exosomal miRNAs also promote the adaptive survival and proliferation of tumor cells through their influence on tumor lesions *via* the circulatory system. Consequently, cardio-miRNAs may negatively affect cancer therapy, even potentially promoting tumor growth, resulting in unfavorable outcomes for patients with concomitant tumors (163). CVD-associated miRNAs may regulate tumor progression through multiple pathways, such as those associated with phosphatase and tensin homolog (PTEN)/phosphoinositide 3-kinase (PI3K)/protein kinase B (AKT), Wnt/β-catenin, NF-κB, and apoptosis. Specifically, the PTEN/PI3K/AKT pathway is known to play a crucial role in controlling the cell cycle, cell proliferation, and the progression of tumors. PTEN suppresses tumor progression by blocking the PI3K/AKT signaling pathway, which is necessary for the survival of tumor cells. Accordingly, the levels of expression and functional effectiveness of PTEN are crucial in determining the fate of tumor cells. In contrast, some cardio-miRNAs, including miR-21, miR-19a, miR-92a/b, miR-25, miR-106b, miR-130b, miR-146b, and miR-210, contribute to tumor growth by directly targeting PTEN and counteracting its inhibitory effect on the PI3K/AKT pathway (164–184). Furthermore, miR-92b also targets disabled homolog 2 interacting protein (Dab2IP), while miR-146 targets tumor necrosis factor receptor-associated factor 6 (TRAF6) and tripartite motif-containing protein 2 (TRIM2), thereby weakening the suppressive effect of the PI3K/AKT signaling pathway and promoting tumor progression. Wnt family proteins are involved in the regulation of cell proliferation, adhesion, migration, and differentiation through β-catenin. Abnormal Wnt/β-catenin signaling can result in tumor cell growth and adaptive survival. MiR-27a enhances the proliferation of triple-negative breast cancer (TNBC) cells by targeting glycogen synthase kinase-3 beta (GSK-3β), leading to the release of β-catenin and its translocation into the nucleus. Additionally, miR-19a inhibits the interaction between SMAD2 and β-catenin, thus fostering tumor progression. The NF-κB pathway also plays a significant role in the regulation of cellular physiology and pathological metabolism. This pathway is highly activated in most cancer cells, promoting their proliferation and survival, which, in turn, drives tumor metastasis and angiogenesis (185–187). Circulating miRNAs secreted by the hearts of individuals with CVD can impact the management of concurrent tumors. For instance, miR-16, miR-150, and miR-423 indirectly stimulate the NF-κB pathway by specifically targeting lactate dehydrogenase-A (LDH-A), forkhead box protein O4 (FOXO4), and TNFAIP3-interacting protein 2 (TNIP2), consequently facilitating cancer advancement (188–190). Similarly, by targeting suppressor of cytokine signaling 1 (SOCS1) and TNIP1, miR-210 indirectly activates the NF-κB pathway, which promotes prostate cancer progression and metastasis (191). Apoptosis is an important mechanism for maintaining tissue homeostasis and eliminating damaged cells. Cancer cells must suppress the apoptosis signaling pathway to achieve adaptive proliferation or survival. MiRNAs play an important role in the regulation of apoptosis, affecting the expression levels of both pro- and antiapoptotic genes. For example, miR-21 and miR-25 influence apoptosis-related signal transduction by attenuating Fas ligand (FASL)/tumor necrosis factor-related apoptosis-inducing ligand (TRAIL) (192, 193) (**Figure 7**). In conclusion, miRNAs represent a pathological link between CVD and cancer.

### 4.2 Myocardial infarction promotes cancer through immune reprogramming

CVD can promote cancer cell proliferation and cancer progression through immune reprogramming. For example, Koelwyn et al. (194) found that MI can accelerate the development of breast cancer through innate immune reprogramming and that MI increases the levels of circulating Ly6C^hi^ monocytes and contributes to their recruitment to tumor tissue. Meanwhile, the consumption of these cells also counteracts MI-induced tumor growth. Monocytes and monocyte-derived macrophages play a crucial role in promoting tumor development by supporting tumor immune evasion, angiogenesis, tumor cell proliferation, and invasion (194). In a study where syngeneic E0771 cancer cells were implanted in C57BL/6J mice, the researchers found that MI accelerated tumor growth and increased tumor cell proliferation within the tumor margins. Further analysis using flow cytometry revealed higher proportions of CD45^+^ cells in the tumors of MI-treated mice, along with an increase in the number of CD11b^+^Ly6G^-^Ly6C^hi^ monocytes resembling monocyte myeloid-derived suppressor cells. These monocytes may impede T-cell infiltration and anti-tumor immune responses. Moreover, tumors from MI mice contained fewer T lymphocytes (CD3^+^ cells) and a higher percentage of immunosuppressive CD4^+^FoxP3^+^ T regulatory (T_reg_) cells compared with sham-operated animals, indicative of active immune suppression. Overall, the results of this study suggested that the accelerated tumor growth observed in MI-treated mice was linked to immune suppression within the tumor microenvironment.

High levels of circulating monocytes are known to be associated with a poor prognosis in several cancers (194, 195). Koelwyn et al. (194) further found that in E0771 tumor-bearing mice, MI treatment resulted in a sustained increase in circulating Ly6C^hi^ monocytes compared with that observed in animals subjected to the sham operation. This may be due to the effect of MI on systemic hematopoiesis, as the number of common myeloid progenitor cells (Ly6C^hi^ monocyte precursors) was higher after MI than after the sham operation. The authors then investigated whether MI promoted the recruitment of Ly6C^hi^ monocytes to tumors by performing adoptive transfer experiments. Their findings suggested that the upregulation of the C-X-C motif chemokine 13 (CXCL13)/C-X-C chemokine receptor type 5 (CXCR5) axis could potentially explain the heightened recruitment of Ly6C^hi^ monocytes to tumors. Subsequently, to verify whether MI-induced tumor growth requires increased Ly6C^hi^ monocyte availability and recruitment, a C-C chemokine receptor type 2 (CCR2)-diphtheria toxin receptor mouse model was established, in which monocytes were eliminated 7 days after MI or sham surgery. This depletion abolished the tumor growth-promoting effect of MI. Flow cytometry-based analysis of T-cell populations within tumors revealed a reduction in the number of CCR2^+^ cells, leading to a decrease in the percentage of CD4^+^FOXP3^+^ Treg cells, which are immunosuppressive, and an increase in activated CD8+ cytotoxic T cells expressing granzyme B. Thus, the findings of this study indicated that Ly6Chi monocytes expressing CCR2 play a crucial role in promoting tumor growth in response to MI, and their influx hinders the development of anti-tumor immune responses.

To investigate the impact of MI on monocyte phenotypes within tumors, the researchers first isolated Ly6C^hi^ monocytes from tumors and assessed their ability to modify CD8^+^ T-cell activation and proliferation. Although no significant difference in CD8^+^ T-cell proliferation was noted, Ly6C^hi^ monocytes from MI mice demonstrated a greater capacity to suppress CD8^+^ T-cell activation compared with those from sham-operated animals. Subsequently, the researchers further assessed the role of CD8^+^ T-cell inhibition in MI-induced tumor progression by administering anti-CD8 or control IgG antibodies to E0771 tumor-bearing mice. They discovered that targeting CD8^+^ cells with an anti-CD8 antibody hastened tumor growth in sham-operated mice but did not affect tumor progression in MI-operated animals. These findings indicated that the pro-tumor effects of MI were partly mediated by a dysfunctional CD8^+^ T-cell response. The authors then examined whether the immunosuppressive gene expression profile observed in Ly6C^hi^ tumor monocytes was also present in circulating and bone marrow-resident monocytes before tumor infiltration, and confirmed that it was. This implied that MI induced systemic monocyte reprogramming, which persisted post-tumor development. Moreover, following MI, chromatin accessibility related to immune responses, inflammation, lymphocyte activation, and cytokine production in myeloid monocytes was diminished, as identified through high-throughput chromatin analysis. Furthermore, binding sites for the transcription factors PU.1, cyclin-E-binding protein 1 (CEBP), and interferon regulatory factor 8 (IRF-8), which regulate myeloid cell differentiation, were identified in these inaccessible chromatin regions post-MI. To assess whether changes in the bone marrow environment were responsible for the accelerated tumor growth observed after MI, the researchers transplanted bone marrow from MI-operated or sham-operated mice into wild-type mice. They reported that mice receiving bone marrow from MI donors exhibited faster tumor growth and greater numbers of circulating monocytes when compared with mice receiving bone marrow from sham-operated donors. These results implied that MI can cause long-term chromatin alterations that contribute to the persistence of an immunosuppressive phenotype, thereby accelerating tumor growth (152) (**Figure 6**).

### 4.3 CVD promotes tumor growth by inhibiting the sensitivity of cancer cells to ferroptosis

The diseased heart can facilitate tumor growth by releasing exosomes that inhibit ferroptosis in tumor cells. Ferroptosis is an intracellular iron-dependent form of programmed cell death distinct from apoptosis, necrosis, and autophagy. This process is crucial for tumor suppression and presents novel avenues for cancer treatment (196). Exosomes are small vesicles with diameters ranging from 30-150 nm that are secreted by cells. They carry a variety of macromolecules, such as proteins, RNA, and lipids, and serve as vital agents for intercellular communication. These vesicles can be absorbed by other cells, subsequently discharging their contents. One study found that exosomes secreted by cardiac cells reduced the sensitivity of tumor cells to ferroptosis in the presence of heart disease. Notably, in cardiomyocytes of mice with chronic heart infarction and patients suffering from heart failure, microRNA (miR)-22-3p was shown to be encapsulated in exosomes, and subsequently transferred to tumor cells, showing significant upregulation. This translocation of miR-22-3p led to an attenuated tumor cell response to erastin-induced ferroptotic death, an effect that was achieved through the suppression of its proferroptotic target gene acyl-CoA synthetase long-chain family member 4 (*ACSL4*). Thus, preventing the biosynthesis of miR-22-3p or inhibiting the activation of its target gene, *ACSL4*, shows promise as a novel therapeutic strategy for neutralizing the impact of CVD on tumor progression by halting the exosome-mediated pathological communication between MI and tumor tissue (197) (**Figure 6**).

### 4.4 Post-myocardial infarction heart failure promotes tumor growth via the nerve growth factor-tropomyosin receptor kinase A pathway

One study showed that MI-induced heart failure can promote breast cancer growth *via* the NGF-TRKA pathway. In this work, the investigators generated a syngeneic mouse model of MI followed by the implantation of 4T1 cancer cells 2 weeks after surgery. The results showed that tumor volume was significantly increased in MI mice compared with that in sham-operated animals. To investigate the mechanisms behind tumor growth, the researchers conducted RNA sequencing and found that the PI3K/AKT pathway was upregulated. Furthermore, the levels of phosphorylated tropomyosin receptor kinase A (TRKA), an upstream regulator of the PI3K-AKT signaling pathway, were elevated in tumor tissue of MI mice. Given the vital role of the neurotrophin protein family in PI3K/AKT regulation, the researchers focused on studying neurotrophic factors, particularly nerve growth factor (NGF), a TRKA ligand, and identified a notable rise in serum NGF levels in peripheral blood post-MI. These findings suggested that elevated NGF levels in the myocardium and circulation contribute to TRKA phosphorylation in tumor tissue, potentially hastening tumor growth in mice with MI-induced heart failure. Subsequent analysis revealed that stimulation with NGF significantly increased the proliferation rate of 4T1 cells and induced TRKA, AKT, and ERK phosphorylation. Inhibiting TRKA in 4T1 cells restricted the NGF-induced increase in the number of Ki67-positive cells, which was linked to an overall upsurge in cell numbers. These results underscore the critical role of the NGF-TRKA pathway in 4T1 cell proliferation and suggest that TRKA inhibition can curb MI-induced tumor growth. Additionally, the above observations imply that targeting TRKA represents a promising approach for treating breast cancer growth in patients with MI-related heart diseases, including heart failure (198) (**Figure 6**).

## 5 Outlook

The study of the relationship between cancer and CVD is a current topic of great interest in the medical field. An increasing number of studies have shown that there is a close association between the two conditions. In summary, cancer may promote CVD through several mechanisms, including shared risk factors (20, 199), the cardiotoxic effects of inflammatory states associated with malignant tumors (54), and cancer therapies that either directly harm key cardiac structures or indirectly promote atherosclerosis, thereby increasing the risk for CVD (62). Similarly, CVD can promote tumor progression through the secretion of factors and genetic reprogramming. The continued in-depth exploration of the interactions between cancer and CVD will help improve the prevention, diagnosis, and treatment of these diseases in clinical practice.

From an epidemiological perspective, the occurrence of CVD in individuals with cancer is notably greater than in the overall population, and the existence of CVD is linked to an increased occurrence of cancer and worse outcomes among cancer patients. Therefore, understanding cancer and CVD pathogenesis and the associated risk factors helps in early intervention and the prevention of related complications. Moreover, cancer treatment leads to decreased heart function and arrhythmia, among other complications. Accordingly, research on how to minimize cardiovascular toxicity while ensuring the effectiveness of tumor therapy is urgently required. In addition, there may be common pathogenesis and biomarkers between cancer and CVD. Through the in-depth study of the association between the two, it may be possible to identify common biomarkers that can be used for the diagnosis and treatment of tumors as well as for the screening and prediction of CVD. This will greatly facilitate the development of personalized medicine and provide patients with more precise and effective treatment plans. We are now at a stage where the association between these two diseases requires extensive exploration; the two main areas for future research are outlined in **Table 2**. Overall, studying the relationship between tumors and CVD has important clinical significance, and is expected to contribute substantially to improving patient survival.

**Table 2:** Future research areas on the relationship between CVD and tumors.

Research areas	Investigation route
Epidemiology	Through large-scale epidemiological surveys, extensive population data is collected to analyze the relationship between cancer patients and those with cardiovascular diseases (CVDs). This can help identify potential risk factors, incidence rates
Animal Models and Cell-based Experiments	Employing animal and cellular models, the occurrence and development of cancers and CVDs can be simulated and the pathophysiological mechanisms can be explored. This can help validate the correlations observed in human studies and delve deeper into possible causal relationships and mechanisms of action

## Glossary

ACSL4: Acyl-CoA synthetase long-chain family member 4
AKT: Protein kinase B
AP-1: Activator protein-1
APL: Antiphospholipid antibody
APS: Antiphospholipid syndrome
ATF3: Activating transcription factor 3
ATP: Adenosine triphosphate
BCR-ABL: Breakpoint cluster region-Abelson 1
β2GPI: Beta-2 glycoprotein I
BRAF: v-Raf murine sarcoma viral oncogene homolog B1
Ca^2+^: Calcium ions
CAR T cell: Chimeric antigen receptor T cell
CCR2: C-C chemokine receptor type 2
CD28: Cluster of differentiation 28
CEBP: Cyclin-E-binding protein 1
cMSC: Cardiac mesenchymal stromal cell
CRS: Cytokine release syndrome
CsA: Cyclosporine A
CTGF: Connective tissue growth factor
CTLA-4: Cytotoxic T lymphocyte antigen-4
CVD: Cardiovascular disease
CXCL13: C-X-C motif chemokine 13
CXCR5: C-X-C chemokine receptor type 5
Dab2IP: Disabled homolog 2 interacting protein
DOX: Doxorubicin
FABP: Fatty acid-binding protein
FAP: Fibroblast-activating protein
FASL: Fas ligand
FATP: Fatty acid transporter protein
FFA: Free fatty acid
FOXO4: Forkhead box protein O4
GSK-3β: Glycogen synthase kinase-3 beta
HER2: Human epidermal growth factor receptor
IL-10: Interleukin-10
IL-1β: Interleukin-1 beta
IL-33: Interleukin-33
IL-6: Interleukin-6
IRF-8: Interferon regulatory factor 8
LDH-A: Lactate dehydrogenase-A
LDL: Low-density lipoprotein
LVSD: Left ventricular systolic dysfunction
mAb: Monoclonal antibody
MAPK: Mitogen-activated protein kinase
MI: Myocardial infarction
mMDSC: Monocyte myeloid-derived suppressor cell
MPO: Myeloperoxidase
mTOR: Mammalian target of rapamycin
NF-κB: Nuclear factor-kappa B
NGF: Nerve growth factor
NOX: NADPH oxidase
PD-1: Programmed death protein-1
PD-L1: Programmed death-ligand 1
PI3K: Phosphatidylinositol-3-kinase
PTEN: Phosphatase and tensin homolog
ROS: Reactive oxygen species
rpS6: Ribosomal protein S6
RUNX2: Runt-related transcription factor 2
SMAD1/5: Suppressor of mothers against decapentaplegic 1/5
SMAD2/3: Suppressor of mothers against decapentaplegic 2/3
SMAD6: Suppressor of mothers against decapentaplegic 6
SOCS1: Suppressor of cytokine signaling 1
TGF-β: Transforming growth factor-beta
TNBC: Triple-negative breast cancer
TNF-α: Tumor necrosis factor-alpha
TNIP1: TNFAIP3 interacting protein 1
TNIP2: TNFAIP3 interacting protein 2
TOP2B: Topoisomerase-2-beta
TRAF6: Tumor necrosis factor receptor-associated factor 6
TRAIL: Tumor necrosis factor-related apoptosis-inducing ligand
TRIM2: Tripartite motif-containing protein 2
TRKA: Tropomyosin receptor kinase A
VEGF: Vascular endothelial growth factor
